# Guidelines, position statements, and advisories for the primary prevention of type 2 diabetes, hypertension, and cardiovascular disease in rural populations: A systematic review protocol

**DOI:** 10.1371/journal.pone.0288116

**Published:** 2023-06-29

**Authors:** Hanieh Sadat Tabatabaei Yeganeh, Larry J. Prokop, Shangwe A. Kiliaki, Karthik Gnanapandithan, Mohammed Yousufuddin, Adrian Vella, Victor M. Montori, Sagar B. Dugani

**Affiliations:** 1 Division of Endocrinology, Diabetes & Metabolism, Mayo Clinic College of Medicine, Rochester, MN, United States of America; 2 Mayo Clinic Libraries, Rochester, MN, United States of America; 3 Division of Hospital Internal Medicine, Mayo Clinic, Rochester, MN, United States of America; 4 Division of Hospital Internal Medicine, Mayo Clinic, Jacksonville, FL, United States of America; 5 Division of Hospital Medicine, Mayo Clinic Health System, Austin, MN, United States of America; 6 Knowledge and Evaluation Research Unit, Mayo Clinic, Rochester, MN, United States of America; 7 Division of Health Care Delivery Research, Robert D. and Patricia E. Kern Center for the Science of Health Care Delivery, Mayo Clinic, Rochester, MN, United States of America; FMUP: Universidade do Porto Faculdade de Medicina, PORTUGAL

## Abstract

**Introduction:**

Globally, noncommunicable diseases (NCDs), which include type 2 diabetes (T2D), hypertension, and cardiovascular disease (CVD), are associated with a high burden of morbidity and mortality. Health disparities exacerbate the burden of NCDs. Notably, rural, compared with urban, populations face greater disparities in access to preventive care, management, and treatment of NCDs. However, there is sparse information and no known literature synthesis on the inclusion of rural populations in documents (i.e., guidelines, position statements, and advisories) pertaining to the prevention of T2D, hypertension, and CVD. To address this gap, we are conducting a systematic review to assess the inclusion of rural populations in documents on the primary prevention of T2D, hypertension, and CVD.

**Methods and analysis:**

This protocol follows the Preferred Reporting Items for Systematic Reviews and Meta-Analyses guidelines. We searched 19 databases including EMBASE, MEDLINE, and Scopus, from January 2017 through October 2022, on the primary prevention of T2D, hypertension, and CVD. We conducted separate Google^®^ searches for each of the 216 World Bank economies. For primary screening, titles and/or abstracts were screened independently by two authors (databases) or one author (Google^®^). Documents meeting selection criteria will undergo full-text review (secondary screening) using predetermined criteria, and data extraction using a standardized form. The definition of rurality varies, and we will report the description provided in each document. We will also describe the social determinants of health (based on the World Health Organization) that may be associated with rurality.

**Ethics and dissemination:**

To our knowledge, this will be the first systematic review on the inclusion of rurality in documents on the primary prevention of T2D, hypertension, and CVD. Ethics approval is not required since we are not using patient-level data. Patients are not involved in the study design or analysis. We will present the results at conferences and in peer-reviewed publication(s).

**Trial registration:**

**PROSPERO Registration Number:** CRD42022369815.

## Introduction

Noncommunicable diseases (NCDs), including type 2 diabetes (T2D), hypertension, and cardiovascular disease (CVD), are major contributors to global morbidity and mortality [1,2]. Globally, 537 million people have T2D, 1.39 billion have hypertension, and over 18.6 million deaths annually are attributed to CVD. Of these deaths, 85% can be attributed to coronary heart disease and cerebrovascular diseases. These conditions disproportionately impact low- and middle-income economies than high-income economies [3–6].

The global burden of T2D, hypertension, and CVD is exacerbated by health disparities, in particular, for rural populations. A systematic review of 109 population-based surveys showed an increase in the prevalence of diabetes in rural populations worldwide, with differences based on economic status. In high-income-economies, the prevalence of diabetes in rural populations increased 1.7-fold from 8.2% (95% confidence interval, 6.0% to 10.4%) in 1985–1989 to 14.3% (8.7% to 20%) in 2005–2011. However, in low-middle-income economies, the prevalence of diabetes in rural populations increased 4.2-fold from 1.8% (1.0% to 2.6%) in 1985–1989 to 7.5% (5.6% to 9.5%) in 2005–2011 [7]. A pooled analysis of studies from 42 countries showed that rural, compared with urban, populations had a lower relative risk of meeting performance measures for diabetes diagnosis and treatment (15% to 30% lower relative risk) and of achieving glycemic control (14% lower relative risk) [8].

Similar to diabetes, the rural burden of hypertension has increased. In a systematic review of 299 population-based surveys in low-middle-income countries, from 1990 to 2020, the prevalence of hypertension increased in urban and rural areas, with a stronger trend in rural areas [9]. The urban-rural difference in prevalence, from 1990 to 2020, was 2.7% (95% confidence interval, 1.4% to 4.1%) in low-middle-income countries and −1.2% (95% confidence interval, −2.7% to 0.3%) in upper-middle-income countries [9].

Similarly for CVD, in 2017, data from the US Centers for Disease Control and Prevention (CDC) showed a higher prevalence of heart disease in US rural (14.2%), compared with small metropolitan (11.2%) and urban (9.9%), residents [10]. In a longitudinal analysis from 1999 to 2017, the age-adjusted cardiovascular mortality rate in 1999, 2011, and 2017, was examined and found to be higher in rural vs. nonrural populations. In 2017, the age-adjusted cardiovascular mortality rate per 100,000 population was 251.4 (rural areas), 221.8 (medium and small metropolitan areas), and 208.6 (large metropolitan areas) [11]. The risk for NCDs may also be mitigated by effective primary prevention, which may require strategies to optimize individual and community factors that contribute to the burden of NCDs [12,13].

International comparison of the NCD burden in rural areas is challenged by the non-standardized definitions of rurality globally and within nations. In the US, there are at least three definitions of rurality. According to the US Office of Management and Budget, counties may be classified as metropolitan (core urban area and population ≥50,000), micropolitan (no core urban area and population 10,000 to 50,000), or neither. This classification categorizes 15% to 20% of the population and 75% of the land as rural [14]. According to the 2013 National Center for Health Statistics system (NCHS), counties may be categorized as large central metro (most urban), large fringe metro, medium metro, small metro, micropolitan, and noncore (most rural) [15]. Alternative definitions are based on census tracts (sub-county classification) rather than counties, such as rural-urban commuting area codes, which are used by the Federal Office of Rural Health Policy [16]. At the state and federal levels, different definitions have been used for legislation, research, and data collection [17]. At the international level, there are efforts to harmonize the definition and facilitate research on rural health [18]. The lack of standardization of definitions/descriptions for rurality makes it challenging to identify, develop, and implement prevention strategies. In rural areas, the prevalence of NCDs may be influenced by multiple factors including health care resources, lifestyle, and socioeconomic factors. Implementing targeted primary prevention strategies in rural areas may significantly reduce the burden of NCDs and improve health in rural populations.

In addition to defining rurality by population and/or proximity to core urban areas, proxy measures, which may be more prevalent in rural vs. nonrural areas, may also be used to study rurality [19,20]. In this context, social determinants of health (SDOH) may highlight factors that contribute to differences in rural and nonrural populations. The World Health Organization (WHO) has described ten categories of SDOH that may impact health equity: income and social protection, education, unemployment and job insecurity, working conditions, food insecurity, housing, access to basic amenities and the environment, early childhood development, social inclusion and non-discrimination, structural conflict, and availability of reasonably priced, high-quality healthcare services. These categories are not specific to rurality but may be considered while assessing rural-nonrural disparities [21].

Improvement in the primary prevention of T2D, hypertension, and CVD in rural populations may benefit people, communities, and healthcare organizations, and guide the development of tailored interventions [22,23]. To our knowledge, there are no published systematic reviews of guidelines, position statements, or advisories on rurality and the primary prevention of these NCDs. To address this knowledge and practice gap, we developed a protocol for a systematic review to describe the incorporation of rurality in guidelines, position statements, and advisories. The primary outcome of the systematic review is incorporation of rurality and/ or SDOH in guidelines, position statements, and advisories for the primary prevention of T2D, hypertension, and CVD. The protocol is designed to evaluate documents from all economies, which will identify areas for improvement and inform the development of targeted primary prevention.

## Methods and analysis

### Standards

The study protocol is reported following the Preferred Reporting Items for System Reviews and Meta-Analyses Protocols (PRISMA-P) guidelines. The systematic review will be conducted and reported using PRISMA guidelines.

### Protocol and registration

We searched electronic databases for documents (i.e., guidelines, position statements, and advisories) on rurality and the primary prevention of T2D, hypertension, and CVD. The search covered the period from January 1, 2017, to October 24, 2022. We focused on the last five years in order to identify recent literature on these topics. The search strategy was designed and conducted by an experienced medical librarian with input from the study authors. Controlled vocabulary supplemented with keywords was used to search for documents on rurality and the primary prevention of T2D, hypertension, and CVD.

### Information sources

We searched the following electronic databases: Ovid MEDLINE and Epub Ahead of Print, In-Process & Other Non-Indexed Citations, and Daily, Ovid EMBASE, Ovid Cochrane Central Register of Controlled Trials, Ovid Cochrane Database of Systematic Reviews, Ovid ACP Journal Club, Ovid Cochrane Clinical Answers, Ovid Cochrane Methodology Register, Ovid EBM Reviews Health Technology Assessment, Ovid EMB Reviews, NHS Economic Evaluation Database [24], Scopus, Web of Science [25], Guidelines International Network (GIN) Guidelines Central [26], the National Health & Medical Research Council Database [27], National Institute for Health and Care Excellence (NICE) [28], Scottish Intercollegiate Guidelines Network (SIGN) [29], Canadian Medical Association Clinical Practice Guidelines Infobase [30], and ECRI institute/Guidelines.gov. [31] The Scopus search strategy is reported in the online supplementary appendix A in S1 File, and other search strategies will be reported with the systematic review.

In addition to electronic databases, we searched World Bank economy-based documents using Google^®^ search engine to identify documents published outside the peer-reviewed literature. The search was conducted using (e.g., diabetes and [guideline or advisory or "position statement"]) and individual economy name with a date filter of January 1, 2017 to October 24, 2022. The list of 216 economies was obtained from The World Bank portal [32].

The study protocol is registered online with PROSPERO (CRD42022369815).

### Eligibility criteria

We included English-language documents on primary prevention strategies in human participants aged 18 years or older. These documents included guidelines, position statements, and advisories from governments and professional societies. We excluded the following document types: conference abstracts, editorials, research letters, viewpoints, opinions, case reports, case series, meta-analysis, undergraduate or graduate level theses, qualitative research studies, and non-peer reviewed research articles such as book chapters. We excluded documents exclusively on type 1 diabetes mellitus, gestational diabetes mellitus, Coronavirus Disease 19 (COVID-19) and on the secondary prevention of T2D, hypertension, and CVD.

### Interventions

This study will not evaluate or summarize the efficacy of interventions.

### Primary screening

Documents were identified from electronic databases; duplicate documents were excluded and the remaining exported to Covidence^®^, which is a primary screening and data extraction tool for systematic reviews [33]. Primary screening consisted of screening abstracts and/or titles based on the study selection criteria and was completed independently by two authors. For economy-based documents, the Google^®^ search was limited to the first five pages, and primary screening was completed by one author. Disagreement between authors was resolved through discussion between the authors, and if required, through consultation with a third author.

### Secondary screening

Following primary screening, we will conduct full-text screening (i.e., secondary screening) of documents selected during primary screening. One author will review the bibliography of documents selected after secondary screening to identify relevant documents as previously described and in accordance with Cochrane recommendations [34,35]. This process will be repeated until no new documents are identified.

### Data extraction and synthesis

Data extraction will be conducted independently by two authors using a standardized data extraction form. Disagreement between authors will be resolved through discussion between the authors, and if required, through consultation with a third author. The following data will be extracted: first author and year of publication, study location, document source (i.e., government or non-government source), definition of T2D, definition of rurality, inclusion of rurality, information on SDOH, definitions for SDOH, and recommendations to address SDOH. For US documents, we will extract information on authors’ rurality based on the county in which their institution is located and categorize county-level rurality using the NCHS 2013 classification system [15].

### T2D, hypertension, and CVD

We will include documents on the primary prevention of T2D, hypertension, and CVD (i.e., myocardial infarction, stroke, and peripheral arterial disease). We will report the definition and diagnostic criteria for T2D, hypertension, and CVD in the document.

### Rurality

There is no universal definition for rurality. If available, we will report the definition provided in the document. For U.S. based studies, we will categorize rurality based on the National Center for Health Statistics (NCHS) 2013 classification of county-level rurality: large central metro counties (most urban), large fringe metro counties, medium metro counties, small metro counties, micropolitan counties, and noncore counties (most rural) [15].

### Social determinants of health

Documents may not explicitly mention rurality but rather include measures associated with rurality. Therefore, we will also extract data on social determinants of health (SDOH) that may be associated with rurality. We will use the WHO SDOH list, published in 2011, because these SDOH may have informed guidelines, position statements, and advisories published between 2017 and 2022. The SDOH reported by the World Health Organization were (a) Income and social protection, (b) Education, (c) Unemployment and job insecurity, (d) Working life conditions, (e) Food insecurity, (f) Housing, basic amenities and the environment, (g) Early childhood development, (h) Social inclusion and non-discrimination, (i) Structural conflict, and (j) Access to affordable health services of decent quality. We will provide a free-text option ‘Other’ to extract other reported SDOH [21].

### World Bank economies

We will summarize evidence based on economies using The World Bank categories of high-income economy, upper-middle-income economy, lower-middle-income economy, and low-income economy [32].

### Risk of bias and quality assessment

The study will include guidelines, position statements, and advisories, which may be reported according to different local, regional, or national standards. Given our focus on summarizing evidence from the documents, we will not conduct a risk of bias assessment or assess the quality of the documents.

### Meta-analysis

This study will not involve a meta-analysis; therefore, we do not have formal controls or sample size calculations. However, we will group and qualitatively compare documents based on economy status. Furthermore, for US documents, we will categorize according to county-level rurality of authors’ institutions.

### Current study status

At the time of preparing the manuscript, primary screening has been completed by two authors (electronic databases) or one author (Google^®^ search). Duplicate documents were identified and excluded, and a final list of documents for secondary screening was prepared. The standardized data extraction form was designed for data extraction following secondary screening. The study team has initiated secondary screening for T2D documents and plans to subsequently apply the same procedure for hypertension and CVD documents. Data extraction for all screened documents will likely be completed in April 2023 (diabetes) and June 2023 (hypertension and CVD).

### Ethics and dissemination

The use of publicly available documents that include aggregate-level, nonidentifiable participant data did not require consent from the Institutional Review Board. The complete search strategy will be published with the systematic review. The results will be presented at conferences and through peer-reviewed publication(s).

### Patient and public involvement

There will be no patient or public participation in the design, conduct, reporting, or dissemination of the study.

## Discussion

Globally, rural populations experience health disparities, in particular, for NCDs including T2D, hypertension, and CVD. To our knowledge, this will be the first systematic review to evaluate the inclusion of rural populations in documents on the primary prevention of T2D, hypertension, and CVD. We will use rigorous methodology and report the results according to PRISMA guidelines. By reviewing and summarizing evidence, where available, from all 216 World Bank economies [32], we will identify knowledge gaps and inform future guidelines, position statements, and advisories. Findings from this systematic review may inform individual- and community-level interventions for primary prevention and clinical practice.

Organizations have started to highlight the importance of rurality and SDOH to NCDs. The American Heart Association and American Stroke Association issued a joint presidential advisory emphasizing the importance of well-being of rural populations. The objective of the advisory is to diminish, and ideally eliminate, health inequalities and enable individuals to lead longer, healthier lives [36]. The US Healthy People 2030 highlights SDOH including to “create social, physical, and economic environments that promote attaining the full potential for health and well-being for all [37].” Additionally, the WHO has emphasized that “developing and adapting health service models to meet the needs of rural and remote communities is an international priority given inequities in health outcomes compared with metropolitan counterparts [38,39]”.

We will limit the search to documents published since 2017 to focus on contemporary documents and ensure that findings reflect current knowledge. We will exclude non-English language documents; however, our analysis provides a template for future work that includes documents in other languages. The strategy to restrict to English language documents may not introduce systematic bias, as reported, and previously described [34,40]. The study will have several strengths. We will include documents from 19 databases and from 216 Google® searches corresponding to the World Bank economies [32]. The study will include SDOH, which may influence rural outcomes. Our approach can be extended to other conditions with a high global burden (e.g., hypertension), which we will undertake in the future.

In this context, findings from our systematic review will be informative to professional societies, policymakers, and government organizations. This work may also inform efforts toward achieving the Sustainable Development Goals 2030, which highlight reducing inequities in health and access to healthcare [41]. This is particularly relevant given that T2D, hypertension, and CVD are public health problems, and superimposed rural-urban health disparities will exacerbate the burden of morbidity and mortality on rural populations.

## Supporting information

S1 Checklist Reporting checklist for protocol of a systematic review and meta analysis. (DOCX)

S1 File Supporting document, appendix A. (DOCX)

## References

[1] Global health estimates: Leading causes of death [Internet]. [cited 2023 Jan 12]. Available from: https://www.who.int/data/gho/data/themes/mortality-and-global-health-estimates/ghe-leading-causes-of-death.

[2] Diabetes [Internet]. [cited 2023 Jan 12]. Available from: https://www.who.int/health-topics/diabetes#tab=tab_1.

[3] Diabetes Facts & figures [Internet]. [cited 2023 Jan 12]. Available from: https://idf.org/aboutdiabetes/what-is-diabetes/facts-figures.html.

[4] What is CVD?—World Heart Day [Internet]. [cited 2023 Jan 12]. Available from: https://world-heart-federation.org/world-heart-day/cvd-causes-conditions/what-is-cvd/.

[5] Dai H, Bragazzi NL, Younis A, et al. Worldwide Trends in Prevalence, Mortality, and Disability-Adjusted Life Years for Hypertensive Heart Disease from 1990 to 2017. Hypertension [Internet]. 2021 Apr 1 [cited 2023 Jan 12];77(4):1223–33. doi: 10.1161/HYPERTENSIONAHA.120.16483 33583201

[6] Mills KT, Stefanescu A, He J. The global epidemiology of hypertension. Nat Rev Nephrol [Internet]. 2020 Apr 1 [cited 2023 Jan 12];16(4):223. doi: 10.1038/s41581-019-0244-2 32024986 PMC7998524

[7] Zabetian A, Sanchez IM, Narayan KMV, et al. Global rural diabetes prevalence: a systematic review and meta-analysis covering 1990–2012. Diabetes Res Clin Pract [Internet]. 2014 [cited 2023 Jan 12];104(2):206–13. doi: 10.1016/j.diabres.2014.01.005 24507869

[8] Flood D, Geldsetzer P, Agoudavi K, et al. Rural-Urban Differences in Diabetes Care and Control in 42 Low- and Middle-Income Countries: A Cross-sectional Study of Nationally Representative Individual-Level Data. Diabetes Care [Internet]. 2022 Sep 1 [cited 2023 Jan 12];45(9):1961–70. doi: 10.2337/dc21-2342 35771765 PMC9472489

[9] Ranzani OT, Kalra A, Di Girolamo C, et al. Urban-rural differences in hypertension prevalence in low-income and middle-income countries, 1990–2020: A systematic review and meta-analysis. PLoS Med [Internet]. 2022 Aug 1 [cited 2023 Jan 12];19(8). doi: 10.1371/journal.pmed.1004079 36007101 PMC9410549

[10] Summary Health Statistics: National Health Interview Survey, 2017. Rockville, MD: Centers for Disease Control and Prevention, National Center for Health Statistics; 2018.

[11] Cross SH, Mehra MR, Bhatt DL, Nasir K, O’Donnell CJ, Califf RM, et al. Rural-Urban Differences in Cardiovascular Mortality in the US, 1999–2017. JAMA [Internet]. 2020 May 12 [cited 2023 Jan 12];323(18):1852–4. doi: 10.1001/jama.2020.2047 32396176 PMC7218488

[12] Saha A, Alleyne G. Recognizing noncommunicable diseases as a global health security threat. Bull World Health Organ [Internet]. 2018 Nov 11 [cited 2023 Jan 12];96(11):792. doi: 10.2471/BLT.17.205732 30455534 PMC6239014

[13] Chand SS, Singh B, Kumar S (2020) The economic burden of non-communicable disease mortality in the South Pacific: Evidence from Fiji [Internet]. 2020 July 23 [cited 2023 Jan 12]; 15(7): e0236068. 10.1371/journal.pone.0236068 PMC7377477 32702003

[14] Defining Rural Population. [Internet]. [cited 2023 Jan 12]. Available from: https://www.hhs.gov/guidance/document/defining-rural-population.

[15] National Center for Health Statistics. 2013 NCHS Urban-Rural Classification Scheme for Counties Series 2, Number 166. 2013. 24776070

[16] Defining Rural Population | HRSA [Internet]. [cited 2023 Jan 12]. Available from: https://www.hrsa.gov/rural-health/about-us/what-is-rural.

[17] Snyder JE, Jensen M, Nguyen NX, et al. Defining Rurality in Medicare Administrative Data. MedCare [Internet]. 2017 [cited 2023 Jan 12];55(12):e164–9. doi: 10.1097/MLR.0000000000000607 29135781

[18] OECD et al. (2021), Applying the Degree of Urbanisation: A Methodological Manual to Define Cities, Towns and Rural Areas for International Comparisons, OECD Regional Development Studies, OECD Publishing, Paris/European Union, Brussels.

[19] Strasser R. Rural health around the world: challenges and solutions. Fam Pract [Internet]. 2003 Aug [cited 2023 Jan 12];20(4):457–63. doi: 10.1093/fampra/cmg422 12876121

[20] Coughlin SS, Clary C, Johnson JA, et al. Continuing Challenges in Rural Health in the United States. J Environ Heal Sci [Internet]. 2019 [cited 2023 Jan 12];5(2):90. 32104722 PMC7043306

[21] Social determinants of health [Internet]. [cited 2023 Jan 12]. Available from: https://www.who.int/health-topics/social-determinants-of-health#tab=tab_1.

[22] US Department of Agriculture Economic Research Service. Rural America at a Glance: 2018 Edition. [cited 2023 Jan 12]. Available from: https://www.ers.usda.gov/webdocs/publications/90556/eib-200.pdf.

[23] Owolabi MO, Yaria JO, Daivadanam M, et al. Gaps in Guidelines for the Management of Diabetes in Low- and Middle-Income Versus High-Income Countries-A Systematic Review. Diabetes Care [Internet]. 2018 May 1 [cited 2023 Jan 12];41(5):1097–105. doi: 10.2337/dc17-1795 29678866 PMC5911785

[24] NHS Centre for Reviews and Dissemination. The NHS Economic Evaluation Database (NHS EED) York: University of York. Effectiveness Matters 6(1). 2002.

[25] Web of Science. Clarivate Accelerating Innovation: Web of Science Database. [Internet]. [cited 2023 Jan 12]. Available from: https://clarivate.com/webofsciencegroup/solutions/web-of-science/.

[26] GIN [Internet]. [cited 2023 Jan 12]. Available from: https://g-i-n.net./

[27] NHMRC [Internet]. [cited 2023 Jan 12]. Available from: https://www.nhmrc.gov.au/.

[28] National Institute for Health and Care Excellence (NICE). [Internet]. [cited 2023 Jan 12]. Available from: National Institute for Health and Care Excellence. (2020). Supporting adult carers (NICE guideline NG150). https://www.nice.org.uk/guidance.

[29] Scottish Intercollegiate Guidelines Network [Internet]. [cited 2023 Jan 12]. Available from: https://www.sign.ac.uk/.

[30] CPG Infobase: Clinical Practice Guidelines. [Internet]. [cited 2023 Jan 12]. Available from: https://joulecma.ca/cpg/homepage.

[31] ECRI Institute to Continue Clinical Guideline Work Shuttered by Federal Government [Internet]. [cited 2023 Jan 12]. Available from: https://www.ecri.org/press/ecri-guideline-website.

[32] World Bank Country and Lending Groups. [Internet]. [cited 2023 Jan 12]. Available from: https://datahelpdesk.worldbank.org/knowledgebase/articles/906519-world-bank-country-and-lending-groups.

[33] Covidence [Internet]. [cited 2023 Jan 12]. Available from: https://www.covidence.org/.

[34] Dugani SB, Melendez APA, Reka R, et al. Risk factors associated with premature myocardial infarction: a systematic review protocol. BMJ Open [Internet]. 2019 Feb 1 [cited 2023 Jan 12]; 9(2). doi: 10.1136/bmjopen-2018-023647 30755446 PMC6377544

[35] Higgins JPT, Thomas J, Chandler J, et al. Chapter 4: Guide to the contents of a Cochrane protocol and review. In: Cochrane Handbook for Systematic Reviews of Interventions. [Internet]. [cited 2023 May 18]. Available from: https://training.cochrane.org/handbook/current/chapter-04.

[36] Harrington RA, Califf RM, Balamurugan A, et al. Call to Action: Rural Health: A Presidential Advisory From the American Heart Association and American Stroke Association. Circulation [Internet]. 2020 Mar 10 [cited 2023 Jan 12];141:E615–44. doi: 10.1161/CIR.0000000000000753 32078375

[37] Social Determinants of Health—Healthy People 2030 [Internet]. [cited 2023 Jan 12]. Available from: https://health.gov/healthypeople/priority-areas/social-determinants-health.

[38] Stockton DA, Fowler C, Debono D, et al. World Health Organization building blocks in rural community health services: An integrative review. Heal Sci Reports [Internet]. 2021 Jun 1 [cited 2023 Jan 12];4(2). doi: 10.1002/hsr2.254 33732894 PMC7942400

[39] Webinar series on Rural Health Equity [Internet]. [cited 2023 Jan 12]. Available from: https://www.who.int/news/item/13-07-2021-webinar-series-on-rural-health-equity.

[40] Morrison A, Polisena J, Husereau D, et al. The effect of English-language restriction on systematic review-based meta-analyses: a systematic review of empirical studies. Int J Technol Assess Health Care [Internet]. 2012 [cited 2023 May 18];28(2):138–144. doi: 10.1017/S0266462312000086 22559755

[41] United Nations Sustainable Development [Internet]. [cited 2023 Jan 12]. Available from: https://sdgs.un.org/goals.

