# Supplementary material for: Guidelines, position statements, and advisories for the primary prevention of type 2 diabetes, hypertension, and cardiovascular disease in rural populations: A systematic review protocol
Source: PLoS One. 2023 Jun 29;18(6):e0288116. doi: 10.1371/journal.pone.0288116 (PMC10309979; doi:10.1371/journal.pone.0288116)
Supplement: S1 File — (DOCX) [file pone.0288116.s002.docx]

**APPENDIX A:** Scopus Database Search Strategy

1 TITLE-ABS-KEY("adult onset diabetes" or "diabetes mellitus type 2" or "diabetes mellitus type II" or "diabetes type 2" or "diabetes type II" or "dm 2" or "insulin independent diabetes" or "ketosis resistant diabetes" or "maturity onset diabetes" or niddm or "non insulin dependent diabetes" or "noninsulin dependent diabetes" or "type 2 diabetes" or "type II diabetes")

2 TITLE-ABS-KEY(((elevated or high) W/3 "blood pressure") or "apparent mineralocorticoid excess syndrome" or hypertension or hypertensive or "liddle syndrome" or "liddles syndrome" or "ocular ischemic syndrome" or "posterior reversible encephalopathy syndrome")

3 TITLE-ABS-KEY(((cerebral or brain) W/3 (insult or insultus or accident* or "blood flow disturbance*" or infarct* or ischem* or ischaem*)) or ((endocardial or subendocardial or angiocardiovascular or cardiovascular or heart or cardiac or cardial or coronary or vascular or myocardial or myocardium or myocard or pericardial or vasculitic or vessel* or aorta or aortic or arteriovenous or artery or arteries or arterial or vein or vein or veins or cerebrovascular) W/3 ("adverse event*" or anomaly or anomalies or abnormalit* or defect or defects or disease* or complication* or disorder* or disturbance* or infection* or inflammation* or lesion* or malformation* or symptom* or syndrome* or event* or deficiency or deformity or dysfunction* or ischemia* or ischaemia* or ischemic or ischaemic or insufficienc*)) or angina or apoplexia or apoplexy or arteriosclerosis or atherosclerosis or "cardiac allograft vasculopath*" or "cardiac arrest" or "cardiac backward failure" or "cardiac death" or "cardiac decompensation" or "Cardiac Failure" or "cardiac incompetence" or "cardiac infarct*" or "cardiac stand still" or "cardiac sudden death" or "cardial decompensation" or "cardial infarct*" or cardiomyopathy or cardiopath* or "cerebral vascular*" or cerebrovascular* or "cerebrum vascular*" or "coronary artery constriction*" or "coronary artery obstruction*" or "coronary artery thrombos*" or "decompensatio cordis" or "heart attack" or "heart backward failure" or "heart death" or "Heart Decompensation" or "heart failure" or "heart incompetence" or "heart infarct*" or "heart micro infarct*" or "heart muscle infarct*" or "insufficientia cardis" or "ischaemic seizure*" or "ischemic seizure*" or "Kounis syndrome" or "Myocardial Failure" or "myocardial hibernation" or "Myocardial Infarct*" or "myocardium infarct*" or "no reflow phenomenon" or "premonitory infarction sign*" or stroke or strokes or "subendocardial infarct*")

4 TITLE(advisory or "consensus document" or "consensus recommendation*" or "consensus report" or "consensus statement" or "consensus study" or "consensus workshop" or guideline* or "position statement" or "Scientific statement")

5 PUBYEAR AFT 2016

6 (1 or 2 or 3) and 4 and 5

7 TITLE-ABS-KEY(newborn* or neonat* or infant* or toddler* or child* or adolescent* or paediatric* or pediatric* or girl or girls or boy or boys or teen or teens or teenager* or preschooler* or "pre-schooler*" or preteen or preteens or "pre-teen" or "pre-teens" or youth or youths) AND NOT TITLE-ABS-KEY(adult or adulthood or adults or centenarian* or elderly or geriatric* or "middle age" or "middle aged" or nonagenarian* or octogenarian* or "old adult*" or "old people" or "old person*" or "older adult*" or "older people" or "older person*" or septuagenarian* or Sextenarian* or "very old")

8 6 and not 7

9 TITLE-ABS-KEY(rural or rurality or socioecologic* or socioeconomic*)

10 8 and 9

11 DOCTYPE(le) OR DOCTYPE(ab) OR DOCTYPE(ed) OR DOCTYPE(bk) OR DOCTYPE(er) OR DOCTYPE(no) OR DOCTYPE(sh)

12 10 and not 11

13 INDEX(embase) OR INDEX(medline) OR PMID(0* OR 1* OR 2* OR 3* OR 4* OR 5* OR 6* OR 7* OR 8* OR 9*)

14 12 and not 13
